# Human Defensins Inhibit SARS-CoV-2 Infection by Blocking Viral Entry

**DOI:** 10.3390/v13071246

**Published:** 2021-06-26

**Authors:** Chuan Xu, Annie Wang, Mariana Marin, William Honnen, Santhamani Ramasamy, Edith Porter, Selvakumar Subbian, Abraham Pinter, Gregory B. Melikyan, Wuyuan Lu, Theresa L. Chang

**Affiliations:** 1 Public Health Research Institute, Rutgers, New Jersey Medical School, The State University of New Jersey, Newark, NJ 07103, USA; cx89@njms.rutgers.edu (C.X.); aw768@njms.rutgers.edu (A.W.); honnenwj@njms.rutgers.edu (W.H.); sr1511@njms.rutgers.edu (S.R.); subbiase@njms.rutgers.edu (S.S.); pinterab@njms.rutgers.edu (A.P.); 2 Department of Pediatrics, Infectious Diseases Emory University, Atlanta, GA 30322, USA; mariana.marin@emory.edu (M.M.); gmelikian@emory.edu (G.B.M.); 3 Department of Biological Sciences, California State University Los Angeles, Los Angeles, CA 90032, USA; eporter@exchange.calstatela.edu; 4 Key Laboratory of Medical Molecular Virology (MOE/NHC/CAMS), School of Basic Medical Science, and Shanghai Institute of Infectious Disease and Biosecurity, Fudan University, Shanghai 200032, China; 5 Department of Microbiology, Biochemistry and Molecular Genetics, Rutgers, New Jersey Medical School, The State University of New Jersey, Newark, NJ 07103, USA

**Keywords:** SARS-CoV-2, defensins, antimicrobial peptides

## Abstract

Innate immunity during acute infection plays a critical role in the disease severity of severe acute respiratory syndrome (SARS) and Middle East respiratory syndrome (MERS), and is likely to contribute to COVID-19 disease outcomes. Defensins are highly abundant innate immune factors in neutrophils and epithelial cells, including intestinal Paneth cells, and exhibit antimicrobial and immune-modulatory activities. In this study, we investigated the effects of human α- and β-defensins and RC101, a θ-defensin analog, on SARS-CoV-2 infection. We found that human neutrophil peptides (HNPs) 1–3, human defensin (HD) 5 and RC101 exhibited potent antiviral activity against pseudotyped viruses expressing SARS-CoV-2 spike proteins. HNP4 and HD6 had weak anti-SARS-CoV-2 activity, whereas human β-defensins (HBD2, HBD5 and HBD6) had no effect. HNP1, HD5 and RC101 also inhibited infection by replication-competent SARS-CoV-2 viruses and SARS-CoV-2 variants. Pretreatment of cells with HNP1, HD5 or RC101 provided some protection against viral infection. These defensins did not have an effect when provided post-infection, indicating their effect was directed towards viral entry. Indeed, HNP1 inhibited viral fusion but not the binding of the spike receptor-binding domain to hACE2. The anti-SARS-CoV-2 effect of defensins was influenced by the structure of the peptides, as linear unstructured forms of HNP1 and HD5 lost their antiviral function. Pro-HD5, the precursor of HD5, did not block infection by SARS-CoV-2. High virus titers overcame the effect of low levels of HNP1, indicating that defensins act on the virion. HNP1, HD5 and RC101 also blocked viral infection of intestinal and lung epithelial cells. The protective effects of defensins reported here suggest that they may be useful additives to the antivirus arsenal and should be thoroughly studied.

## 1. Introduction

Severe acute respiratory syndrome-related coronavirus (SARS-CoV-2), the causative agent of coronavirus disease 19 (COVID-19), has spread worldwide with more than 179 million cases and 3.8 million deaths as of late-June 2021 [1]. The ability of the innate immune response to limit the magnitude of virus replication during acute infection is known to determine the severity of disease outcomes of coronaviruses [2,3,4]. Identification of mucosal factors against SARS-CoV-2 is critical for developing antiviral treatments and therapeutic options for COVID-19. Defensins, antimicrobial peptides that play a crucial role in innate immunity, are abundant in neutrophils and are expressed by epithelial cells at mucosal sites [5,6,7]. Neutrophils have been shown to infiltrate the lung in response to SARS-CoV-2 infection in animal models [8,9,10], suggesting their function in the control of virus replication, although dysregulation of neutrophils may contribute to hyperinflammation in severe COVID-19 diseases [11].

Human defensins are cationic peptides with β-pleated sheet structures that are stabilized by three intramolecular disulfide bonds between cysteine residues [5,6,7]. These defensins are classified into the α-, β- and θ-defensins based on the disulfide links between conserved cysteine residues and their structure [5,6,7]. The effects of defensins on viral infection are defensin-, virus- and cell-type-specific [12,13,14]. Human α-defensins including human neutrophil peptides (HNPs) 1–4 and human α-defensins 5 and 6 (HD5 and HD6), which are mainly produced by neutrophils and intestinal Paneth cells, respectively, exhibit antiviral activities against enveloped and non-enveloped viruses [12]; however, evidence indicates that HD5 and HD6 can also promote viral infectivity of HIV and some adenoviruses [15,16]. Human β-defensins (HBDs) do not appear to affect virions, but these peptides are known to suppress viral replication by altering cellular functions [14]. Theta-defensins (θ-defensins), which are cyclic octadecapeptides expressed in rhesus macaques and baboons, and retrocyclins (RC), which are synthesized based on human θ-defensin pseudogenes, also exhibit antiviral activities [17,18]. In addition to their effects on virus replication, defensins serve as immune modulators to regulate innate and adaptive immunity [19,20]. For example, defensins exhibit inflammatory and anti-inflammatory activities, and recruit and activate T cells and myeloid cells such as monocytes and dendritic cells [19,20]. Administration of rhesus θ-defensin-1 (RTD-1) prevents death in MA15-SARS-CoV-infected mice by reducing pulmonary pathology. The mechanism is thought to involve immune modulation, as RTD-1 does not exhibit anti-SARS-CoV activity in vivo or in vitro [21]. Here, we have determined the effects of various human defensins and a θ-defensin analog RC101 on SARS-CoV-2 infection in vitro and their underlying mechanisms.

## 2. Materials and Methods

### 2.1. Reagents, Plasmids, and Cell Lines

Defensins and their analogs were chemically synthesized, folded and verified as described previously [22]. To construct linear unstructured analogs of HNP1 and HD5, [Abu]HNP1 and [Abu]HD5, the six cysteine residues were replaced by isosteric α-aminobutyric acid (Abu). Recombinant HD5 propeptides (aa 20-94) were synthesized using a baculovirus/insect cell culture system [23]. Recombinant SARS-CoV-2 spike receptor-binding domain (RBD) and hACE2 proteins, expressed in HEK293T cells, were purchased from RayBiotech (Peachtree Corners, GA, USA). The CCF4-AM β-lactamase substrate (GeneBLAzer in vivo detection kit) was purchased from Invitrogen (Carlsbad, CA, USA).

The construct for full-length SARS-CoV-2-Wuhan-Hu-1 surface (spike) (GenBank accession number QHD43416) [24] was codon-optimized for humans and synthesized with Kozak-START GCCACC ATG and STOP codons and with 5′ Nhel/3′Apal sites for subcloning into the pcDNA3.1(+) vector (Thermo Fisher Scientific, Waltham, MA, USA). Plasmids encoding spike proteins of B.1.1.7 and P1 variants were kindly provided by Dennis Burton (The Scripps Research Institute, La Jolla, CA, USA). The packaging HIV-1 pR9ΔEnv (from Chris Aiken at Vanderbilt University), pMM310 expressing β-lactamase-Vpr chimera (BlaM-Vpr, provided by Michael Miller at Merck Research Laboratories and now available from NIH AIDS Research and Reference Program), pMDG-VSV-G plasmid expressing VSV-G (from J. Young at Roche Applied Science, Mannheim, Germany), psPAX2 lentiviral packaging vector and pcRev vector have been described previously [25]. pCAGGS-SARS-CoV-2 S D614G (cat.#156421) and pWPI-IRES-Puro-Ak-ACE2-TMPRSS2 (cat. #154987) expression vectors were obtained from Addgene (Watertown, MA, USA).

HEK293T/17, Vero E6, Caco-2 and A549 cell lines were purchased from American Type Culture Collection (Manassas, VA, USA). HEK293T-hACE2 cells and HeLa-hACE2 cells were kindly provided by Hyeryun Choe (The Scripps Research Institute, Jupiter, Florida, USA) [26] and by Dennis Burton [27], respectively. Huh7.5 was obtained from Dr. Charles Rice (Rockefeller University) through Apath LLC (Brooklyn, NY, USA). To generate Huh7.5-ACE2-TMPRSS2 cells for viral fusion assay, Huh7.5 cells grown at 60% confluency in six-well plates were transduced with single-cycle infectious VSV-G pseudotyped lentiviral viruses (0.5 ng p24/well) encoding for ACE2-TMPRSS2 by centrifugation at 16 °C for 30 min at 1550× *g*. After 24 h transduction, cells were transferred in a 10-cm tissue culture dish in the presence of 2 µg/mL puromycin.

### 2.2. Cell Culture

HEK 293T, HEK293T-hACE2, HeLa-hACE2, Caco-2 and A549 cells were cultured in Dulbecco’s Modified Eagle’s Medium (DMEM) supplemented with 10% heat-inactivated fetal bovine serum. For HEK293T/17 cells, the growth medium was supplemented with 0.5 mg/mL of G418 sulfate (Mediatech, Inc). Huh7.5-hACE2-TMPRSS2 cells were cultured in complete media with 2 µg/mL puromycin.

### 2.3. Production of Pseudotyped Viruses

Replication-defective HIV-1 luciferase-expressing reporter viruses pseudotyped with SARS-CoV-2 S proteins were produced by co-transfection of a plasmid encoding the envelope-deficient HIV-1 NL4-3 virus with the luciferase reporter gene (pNL4-3.Luc.R-E- or pNL4-3.Luc.R+E- from N. Landau, New York University) and a pcDNA3.1 plasmid expressing the SARS-CoV-2 glycoprotein into HEK 293T cells, which were seeded at 6–7 × 10^6^ in a 10-cm dish and cultured overnight, using Lipofectamine 3000 (Thermo Fisher Scientific) as described previously [28]. The supernatant was collected 48 h after transfection and filtered. Virus stocks were analyzed for HIV-1 p24 antigen by the AlphaLISA HIV p24 kit (PerkinElmer). Because serum is known to reduce the effect of defensins on the virion [29,30], viruses were produced under serum-free conditions, or for viruses generated in 10% FBS, these were diluted in serum-free medium (to a final FBS concentration of 3%). To produce serum-free SARS-CoV-2 pseudotyped viruses, the culture media were replaced by DMEM without serum at 24 h after transfection, and the cells cultured for an additional 24 h prior to collecting viruses. Virus stocks contained approximately 200 ng/mL of HIV p24 proteins.

For infection assays, cells plated at 2 × 10^4^ or 5 × 10^4^ cells/well in a 96-well or 48-well plate, respectively, were cultured overnight. Pseudotyped SARS-CoV-2 luciferase reporter viruses were incubated with defensins at 37 °C for 1 h. The defensin-virus mixture was then added to cells. After 1–2 h viral attachment, infected cells were cultured in media with 10% FBS for 48–72 h before measuring luciferase activity using Luciferase Substrate Buffer (Promega Inc). Luciferase activity (relative light units; RLU.) reflecting viral infection was measured on a 2300 EnSpire Multilabel Plate Reader (PerkinElmer, Waltham, MA). Results obtained using different titers of viruses are reported as the average percentage of the control calculated using the formula: (RLU of treated cells / RLUs of untreated cells) × 100.

To generate BlaM-Vpr harboring pseudotyped SARS-CoV-2 viruses for the viral fusion assay, HEK293T/17 cells grown at 75% confluency in a 10-cm dish were transfected with pCAGGS-SARS-CoV-2 S D614G (4 µg), pR9ΔEnv (4 µg), BlaM-Vpr (2 µg) and pcRev (0.5 µg) using the JetPRIME transfection reagent (Polyplus-transfection, Illkirch-Graffenstaden, France). Transfected cells were incubated for 14h at 37 °C and then cultured in a fresh growth medium for an additional 36 h. The viral supernatants were filtered through 0.45 μm polyethersulfone filters (VWR, Radnor, PA, USA) and concentrated 5x using Lenti-X™ Concentrator (Clontech, Mountain View, CA, USA). To produce the transducing VSV-G pseudotyped viruses for generating Huh7.5-hACE2-TMPRSS2 cells, HEK293T/17 cells were transfected with pMDG-VSV-G (1.5 µg), psPAX2 (3 µg) and ACE2-TMPRSS2 expression vectors (4 µg). Viruses were prepared as described above.

### 2.4. Replication-Competent Virus Infection

Replication-competent SARS-CoV-2 viruses expressing mNeonGreen, kindly provided by Pei-Yong Shi at the University of Texas Medical Branch, Galveston, TX, USA, were propagated in Vero E6 cells as described previously [31]. Experiments were performed in a biosafety level 3 laboratory with personal protection equipment including powered air-purifying respirators (Breathe Easy, 3M), Tyvek suits, aprons, sleeves, booties and double gloves. Virus titers were determined by plaque assays in Vero E6 cells as described previously [32]. For the infection assay, Vero E6 cells at 1.5 × 10^4^ cells/well in a black 96-well glass plate (Greiner, Monroe, NC, USA) were incubated overnight. Cells were exposed to viruses (30 μL) with or without defensin treatment at a multiplicity of infection (MOI) of five for 1 h followed by the addition of 100 μL FluoroBrite medium containing 2% FBS. Fluorescence from productive viral infections was monitored at 48 h after infection using a Biotek Cytation 5 multi-mode plate reader.

### 2.5. Viral Attachment Assay

HeLa-hACE2 cells seeded at 5 × 10^4^ per well in 48-well plates were cultured overnight. Pseudotyped viruses were pretreated with or without defensins at 37 °C for 1 h. Cells were incubated with viruses at 4 °C for 2 h, washed with cold PBS three times, and lysed with 100 μL of 1% Triton X-100. Cell-associated HIV p24 was determined by AlphaLISA HIV p24 kit (PerkinElmer).

### 2.6. SARS-CoV-2 RBD and hACE Binding Assay

Greiner Microlon 200 plates (Thermo Fisher Scientific, Waltham, MA, USA) were coated with recombinant SARS-CoV-2 RBD proteins at 50 ng/well in 0.1 M bicarbonate buffer pH 9.6 at 4 °C for 24 h. The plates were blocked with 2% bovine serum albumin in PBS (*w/v*) for 30 min at 37 °C, washed with PBS, and incubated with biotinylated hACE2 proteins (0.1 μg/mL) that were pre-treated with defensins at 37 °C for 1 h. After washing, the bound hACE2 proteins were detected by incubating with alkaline phosphatase-conjugated streptavidin at 37 °C for 1 h. Plates were washed and the binding of hACE2 proteins to SARS-CoV-2 RBD was detected by adding 1 mg/mL of ρ-nitrophenol in DEA buffer at pH 9.8, and measuring the resulting signal at 405 nm.

### 2.7. Virus-Cell Fusion Assay

The virus-cell fusion assay was performed as described previously [33]. Briefly, pseudotyped SARS-CoV-2 viruses containing a β-lactamase-Vpr chimera (BlaM-Vpr) were diluted in the medium without FBS (0.5 ng p24/well), bound to target cells by centrifugation at 16 °C for 30 min at 1550× *g*. Unbound viruses were removed and a growth medium containing or lacking defensins was added. Virus-cell fusion was initiated by incubating the samples at 37 °C for 2 h. Cells were then loaded with the CCF4-AM fluorescent substrate and incubated overnight at 11 °C. The cytoplasmic BlaM activity (ratio of blue to green fluorescence) was measured using the SpectraMaxi3 fluorescence plate reader (Molecular Devices, Sunnyvale, CA, USA).

### 2.8. Cytotoxicity Assay

HEK 293T-hACE2 cells were plated in 96-well plates at 5000 cells per well and then treated with various concentrations of defensins for 24 h. Cell viability was analyzed using MTS-based CellTiter 96^®^ AQueous One Solution Cell Proliferation Assay (Promega, Madison, WI, USA).

### 2.9. Statistical Analysis

Statistical comparisons were performed using a two-tailed Independent-Samples *t*-test; *p* < 0.05 was considered significant. Prism 8 (GraphPad Software, LLC, San Diego, CA, USA) was used for these analyses.

## 3. Results

### 3.1. Human α-Defensins Inhibit SARS-CoV-2 Infection

**Figure 1:**
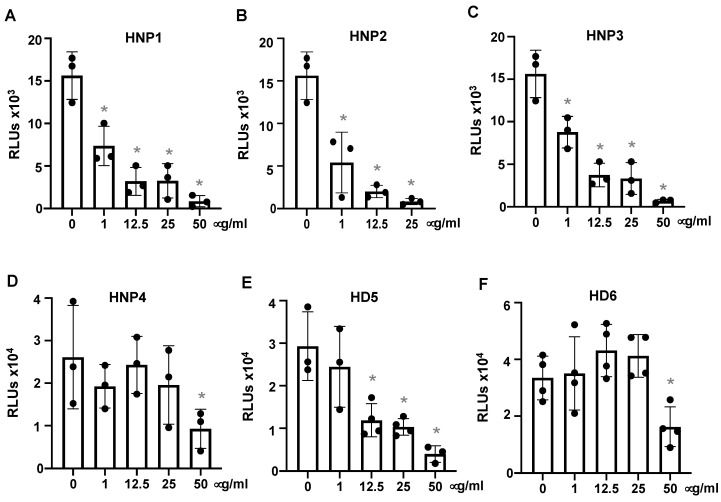
Human α-defensins inhibit infection by pseudotyped SARS-CoV-2 virus. Pseudotyped luciferase reporter virus expressing SARS-CoV-2 S protein was incubated with or without HNPs1-4, HD5 or HD6 at 37 °C for 1 h. Viruses with or without defensin treatment were used to infect HEK293T cells expressing hACE2 (**A**–**F**) as described in the Methods. Infected cells were cultured for three days before measuring luciferase activity. The significance of the differences between defensin-treated virions and mocked-treated controls was calculated by Student’s two-tailed, unpaired *t* = test; * *p* < 0.05. Data are means ± SD and are representative of four independent experiments.

To assess the effect of defensins on SARS-CoV-2 infection, pseudotyped luciferase virus particles expressing SARS-CoV-2 surface (spike, S) proteins were treated with or without defensins for 1 h before infection of HEK293T-hACE2 cells, as described in the Methods. Viral infection was determined by measuring the luciferase activity in cells at day three post-infection. Note that defensins were not added back after attachment. HNPs 1–3 blocked SARS-CoV-2 infection, achieving approximately 50% suppression at 1 μg/mL (290 nM) (Figure 1A–C). Because HNPs 1–3 differ by only a single (N-terminal) amino acid residue, it is not surprising that all three peptides had similar effects. HNP4, a low-abundance peptide in neutrophils and less known for its antiviral activity, was substantially less potent (Figure 1D). Two intestinal defensins (HD5 and HD6) were also studied. HD5 exhibited significant anti-SARS-CoV-2 activity, achieving 60% suppression at 12.5 μg/mL (3.45 μM), whereas HD6 only blocked SARS-CoV-2 infection at the highest concentration tested (50 μg/mL, 13 μM) (Figure 1E,F). The antiviral activity of defensins was not associated with any observed cytotoxicity (Supplemental Figure S1). Of note, the concentrations of HNPs 1–3 and HD5 employed in these experiments were within the physiological ranges of these defensins in neutrophils and in intestinal tissues and lumen [5,34].

**Figure 2:**
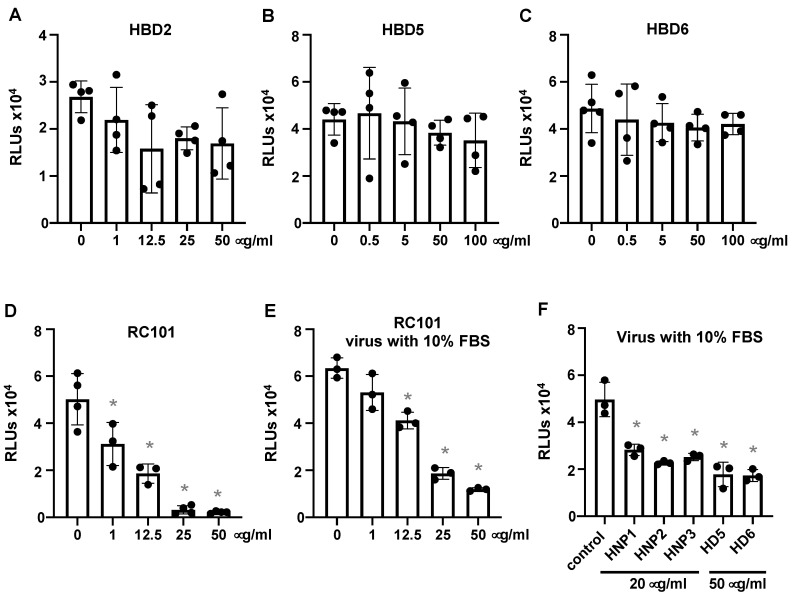
Theta defensin analog RC101 but not HBDs inhibits pseudotyped SARS-CoV-2 infection. Pseudotyped luciferase reporter viruses expressing SARS-CoV-2 S proteins were incubated with or without the indicated concentrations of HBD2, HBD5, HBD6 or RC101 at 37 °C for 1 h followed by infection of HEK293T cells expressing hACE2 (**A**–**D**) as described in the Methods. Infected cells were cultured for three days before measuring luciferase activity. The effect of defensins on the virus in the presence of 10% FBS was also determined (**E**,**F**). Differences between defensin-treated virions and non-treated controls were calculated by Student’s two-tailed, unpaired *t*-test; * *p* < 0.05. Data are means ± SD and are representative of three independent experiments.

Unlike human α-defensins, β-defensins HBD2, HBD5 and HBD6 did not effectively inhibit viral infection (Figure 2A–C). However, the θ-defensin analog RC101 exhibited a similar level of inhibition of SARS-CoV-2 infection (50% inhibition at 6.4 μg/mL, 3.4 μM) as HNPs 1–3 and HD5 (Figure 2D). The human α-defensins as well as RC101 largely maintained their antiviral activities in the presence of serum (Figure 2E,F).

**Figure 3:**
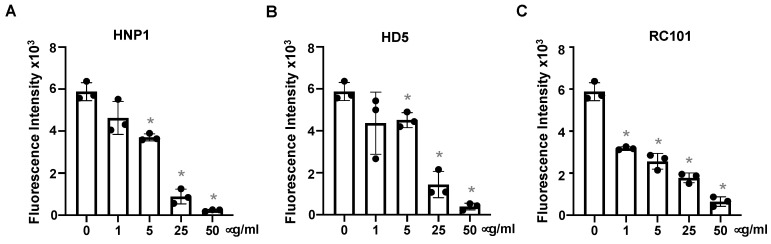
Defensins inhibit infection by replication-competent SARS-CoV-2 viruses. Replication-competent SARS-CoV-2 viruses expressing mNeonGreen (at MOI of five) were incubated with HNP1 (**A**), HD5 (**B**) and RC101 (**C**) at different concentrations and at 37 °C for 1 h before addition to Vero E6 cells for 2 h. Infected cells were then cultured in FluoroBrite media with 10% FBS for two days. The fluorescence from productive viral infections was measured using Biotek Cytation 5. The differences between defensin-treated virions and non-treated controls were calculated by Student’s two-tailed, unpaired *t*-test; * *p* < 0.05. Data are means ± SD and are representative of three independent experiments.

We then determined whether defensins blocked replication-competent SARS-CoV-2 viruses. Replication-competent SARS-CoV-2 viruses expressing mNeonGreen were incubated with HNP1, HD5 and RC101 at different concentrations for 1 h before infection of Vero E6 cells. In agreement with results from pseudotyped SARS-CoV-2 viruses (Figure 1), HNP1, HD5 and RC101 suppressed viral infection in a dose-dependent manner (Figure 3).

**Figure 4:**
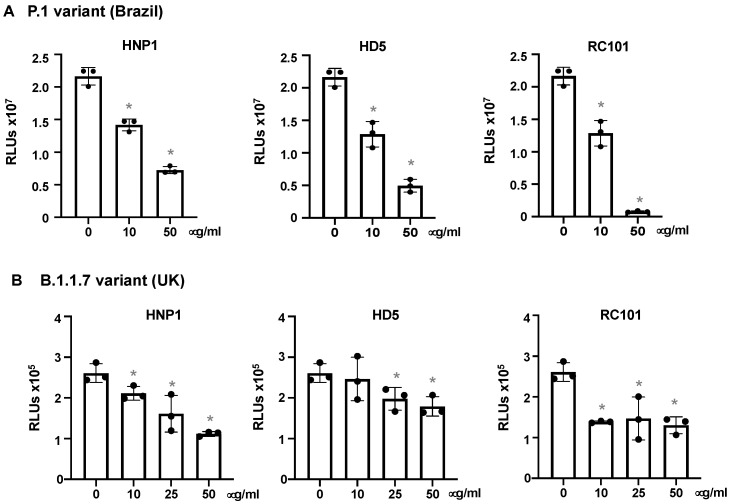
HNP1, HD5 and RC101 inhibit infection by pseudotyped SARS-CoV-2 variants. Pseudotyped luciferase reporter virus expressing SARS-CoV-2 S protein from the P.1 variant (**A**) or the B.1.1.7 variant (**B**) was incubated with or without HNP1, HD5 and RC101 at 37 °C for 1 h. Viruses were added to HeLa-hACE2 cells and incubated at 37 °C for 2 h. Infected cells were cultured for two days before measuring luciferase activity. The significance of the differences between defensin-treated virions and mocked-treated controls was calculated by Student’s two-tailed, unpaired *t*-test; * *p* < 0.05. Data are means ± SD and are representative of three independent experiments.

SARS-CoV-2 variants, including P.1 and B.1.1.7 from Brazil and the United Kingdom, respectively, are highly transmissible and increase the risk of death [35,36,37,38]. We determined the effect of defensins on pseudotyped viruses expressing spike proteins from the P.1 and B.1.1.7 variants. HNP1, HD5 and RC101 suppressed the P.1 variant in a dose-dependent manner (Figure 4A). HNP1, HD5 and RC101 exhibited moderate antiviral activity against the B.1.1.7 variant (Figure 4B). HNP1 and HD5 at 50 μg/mL, which inhibited the earlier Wuhan strain by 96% (Figure 1), suppressed P1 infection by 67% and 72%, respectively, suggesting that the emerged variant was more resistant to host antimicrobial peptides. Similarly, HNP1 and HD5 at 50 μg/mL inhibited B.1.1.7 infection by 58% and 32%, respectively. Although RC101 suppressed infection by both variants, it was also less potent against the B.1.1.7 variant (Figure 4).

### 3.2. Defensins Inhibit Viral Entry

**Figure 5:**
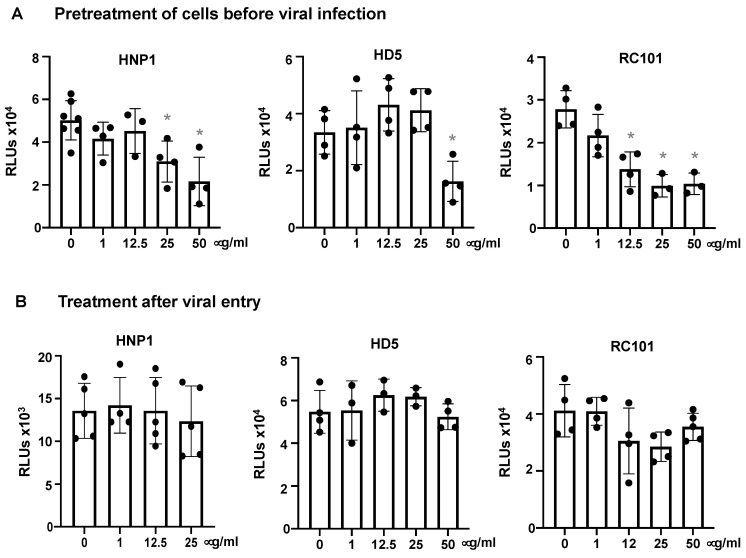
Defensins are ineffective after viral entry. (**A**) To determine the effect of defensins on target cell resistance to infection, HEK293T-hACE2 cells were incubated with defensins in the presence of FBS for 1 h, and then exposed to pseudotyped SARS-CoV-2 luciferase reporter virus without defensins for 2 h. Cells were washed and then cultured in fresh media for three days before measuring luciferase activity. (**B**) To determine the post-entry effect of defensins, HEK293T-hACE2 cells were infected with pseudotyped SARS-CoV-2 viruses for 2 h followed by treatment of infected cells with the indicated concentration of defensins. The differences between defensin-treated samples and untreated controls were calculated by Student’s two-tailed *t*-test; * *p* < 0.05. Data are means ± SD of triplicate samples and are representative of three independent experiments.

We determined whether pre-treatment of cells with defensins provided protection against the virus. Cells were treated with defensins for 1 h, washed and then infected with pseudotyped SARS-CoV-2 virus. Pretreatment of cells with high concentrations of HNP1, HD5 and RC-101 partially blocked viral infection (Figure 5A). To determine whether defensins exhibited an effect after viral entry, cells were infected with pseudotyped SARS-CoV-2 viruses for 1.5 h and then treated with HNP1, HD5 or RC101 for three days. None of the tested defensins affected infection after viral entry (Figure 5B). These results suggested that defensins block SARS-CoV-2 infection at the step of viral entry.

**Figure 6:**
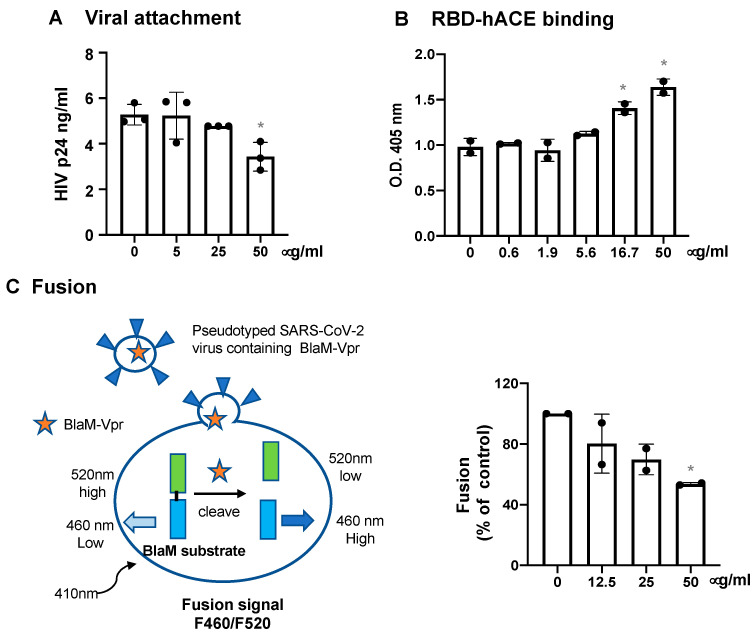
The effect of HNP1 on viral attachment, spike RBD-hACE2 interaction and viral fusion. (**A**) Pseudotyped SARS-CoV-2 viruses were treated with HNP1 at different concentrations for 1 h at 37 °C. The virus-defensin mixture was added to HeLa-hACE2 cells, and cells were incubated at 4 °C for 2 h. After washing off unbound viruses, cells were lysed, and cell-associated HIVp24 was determined as described in the Methods. (**B**) Immobilized spike RBD proteins on the plate were incubated with biotinylated hACE proteins that were pre-treated with defensins at different concentrations for 1 h. After incubation at 37 °C for 1 h, the plate was washed and the bound hACE2 proteins were detected by incubation with alkylate phosphatase (AP)-conjugated streptavidin followed by the AP colorimetric assay as described in the Methods. For panels A and B, the differences between defensin-treated samples and untreated controls were calculated by Student’s two-tailed *t*-test; * *p* < 0.05. Data are means ± SD of triplicate samples and are representative of three independent experiments. (**C**) A schematic of Förster resonance energy transfer (FRET)-based BlaM assay is shown on the left panel. Pseudotyped SARS-CoV-2 viruses containing BlaM-Vpr proteins were incubated with Huh7.5-ACE2-TMPRSS2 cells at 16 °C for 30 min at 1550× *g*. After washing off unbound viruses, cells were treated with HNP1 at different concentrations and incubated at 37 °C for 2 h for viral fusion. The BlaM substrate, CCF4-AM, was added to cells, which were incubated at 11^o^C overnight. The cytoplasmic BlaM activity from viral fusion was determined by measuring the ratio of blue (460 nm) to green (520 nm) fluorescence with excitation at 410 nm. The differences between defensin-treated samples and untreated controls were calculated by Student’s two-tailed *t*-test; * *p* < 0.05. Data are means ± SD of two independent experiments.

The step of viral entry includes viral attachment and the binding of spike proteins through the RBD to hACE2, followed by fusion between the viral and cellular membranes. To assess the effect of HNP1 on viral attachment, pseudotyped SARS-CoV-2 viruses with or without HNP1 pretreatment were incubated with HeLa-hACE2 cells at 4 °C for 2 h. After washing to remove unbound viruses, cells were lysed, and the level of cell-associated HIVp24 was determined. HNP1 did not have a significant impact on viral attachment except that the high concentration (50 μg/mL) of HNP1 suppressed viral attachment by 35% (Figure 6A). We did not find that HNP1 interfered with the interaction between spike RBD and hACE2 proteins (Figure 6B). In contrast, HNP1 at high concentrations (16.7 and 50 μg/mL) slightly promoted the interaction between hACE2 and spike RBD proteins. We then assessed the effect of HNP1 on viral fusion. Pseudotyped viruses expressing SARS-CoV-2 spike D614G proteins (in the UK B.1.1.7 variant) were packaged with BlaM-Vpr. Virus binding to cells was augmented by low-speed centrifugation at 16 °C. After washing off unbound viruses, cells were incubated in medium with or without defensins at 37 °C to allow viral fusion to occur. In the absence of defensins, β-lactamase, released into the cytoplasm from the viruses containing BlaM-Vpr proteins, cleaved the CCF4-AM substrate (Figure 6C, left panel), resulting in an increase in fluorescent signals at 460 nm. We found that HNP1 blocked viral fusion in a dose-dependent manner (Figure 6C, right panel).

### 3.3. Native Structure (Disulfide Bonding) of Defensins Is Required to Inhibit SARS-CoV-2 Infection

**Figure 7:**
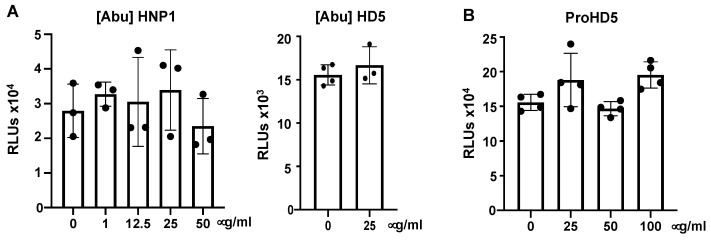
The structure of defensins required for anti-SARS-CoV-2 activity. To determine the effect of linear defensins (**A**) or proHD5 (**B**) on viral infection, pseudotyped SARS-CoV-2 virus was incubated with indicated concentrations of [Abu]HNP1 or [Abu]HD5 for 1 h before infection of HEK293T-hACE2 cells. Infected cells were cultured for three days before measuring luciferase activity. There was no difference between defensin-treated samples and untreated controls as calculated by Student’s two-tailed *t*-test. Data are means ± SD of triplicate samples and are representative of two independent experiments.

The structure of defensins is critical for their effects on viruses [16,39]. To determine whether this was true of SARS-CoV-2 as well, we examined the anti-SARS-CoV-2 activity of [Abu]HNP1 and [Abu]HD5, which are identically charged, but unstructured analogs of HNP1 and HD5 [22]. We treated pseudotyped SARS-CoV-2 virus with these linear defensins before adding the virus to cells and then determined infection at day three post-infection. In contrast to the antiviral activity of HNP1 or HD5 (Figure 1), the linear analogs [Abu]HNP1 and [Abu]HD5 did not have an inhibitory effect on SARS-CoV-2 infection (Figure 7A). These results indicate that the antiviral effect of defensins requires native disulfide bonding and properly folded proteins. HD5 is synthesized as a propeptide (ProHD5) in Paneth cells in the small intestine and in epithelial cells of the genital tract, and is processed to the mature peptide by trypsin or neutrophil proteases [34,40]. The unprocessed pro-HD5 exhibited no anti-SARS-CoV-2 activity (Figure 7B), which is in agreement with prior studies reporting reduced or absent antimicrobial activity of proHD5 compared to processed HD5 [34,40].

### 3.4. The effect of Virus Titers and Cell Types on Defensin-Mediated Viral Infection

**Figure 8:**
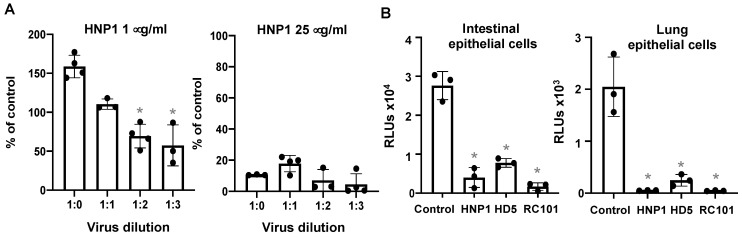
The effect of viral titers and cell types on defensin-mediated SARS-CoV-2 inhibition. (**A**) To determine the effect of virus titers on the antiviral activity of HNP1, indicated dilutions of serum-free pseudotyped SARS-CoV-2 viruses were incubated with 1 μg/mL (*left*) or 25 μg/mL (*right*) HNP1 at 37 °C for 1 h before infection of HEK293T-hACE2 cells. The percentage of control was calculated using the formula: (RLU of defensin-treated samples/RLUs of average of untreated samples at the same virus titer) × 100. (**B**) To determine the effect of defensins on SARS-CoV-2 infection of intestinal and lung epithelial cells expressing endogenous hACE2 receptors, pseudotyped SARS-CoV-2 viruses were incubated with 25 μg/mL HNP1, HD5 or RC101 at 37 °C for 1 h, then added to intestinal CaCo-2 cells (*left*) or lung epithelial A549 cells (*right*) at 37 °C for 1.5 h. After washing off unbound viruses, infected cells were cultured for three days before measuring luciferase activities. The differences between defensin-treated samples and untreated controls were calculated using Student’s two-tailed *t*-test; * *p* < 0.05. Data are means ± SD of triplicate samples and are representative of three independent experiments.

We have previously shown that the direct effect of HNP1 on HIV virions was abolished by an increase in the number of virus particles [30]. To determine whether increasing the virus titer affected the antiviral activity of HNP1, different titers of serum-free pseudotyped SARS-CoV-2 were incubated with 1 or 25 μg/mL HNP1 at 37 °C for 1 h and then added to cells. Viral infection was determined at day three post-infection. At 25 μg/mL, HNP1 inhibited viral infection regardless of the virus titer. At 1 μg/mL, however, HNP1 displayed an inhibitory effect on low concentrations of the virus, but this effect was lost when the viral titer was high (Figure 8A). This result suggest that HNP1 interacts directly with the virus, and is depleted at high viral concentrations (Figure 8A). Finally, we examined whether the SARS-CoV-2 inhibitory effect of defensins was cell-type-dependent. Intestinal epithelial cells (Caco-2) express moderate levels of endogenous hACE2, and lung epithelial cells (A549) have near undetectable levels of hACE2 but a high abundance of SARS-CoV-2 alternative receptors, CD147 and tyrosine-protein kinase receptor UFO (AXL) [28]. We found that HNP1, HD5 and RC101 exhibited anti-SARS-CoV-2 activity in both Caco-2 and A549 cells (Figure 8B), indicating that the inhibitory effect of defensins on viral infection was not receptor-specific or cell-type-dependent.

## 4. Discussion

The ability of the innate immune response to reduce viral replication is known to impact the severity of coronavirus-associated disease outcomes. Defensins are highly abundant antimicrobial peptides produced by neutrophils and epithelial cells, and play a critical role in innate immunity. Here, we showed that human α-defensins including HNPs1–3 and HD5 as well as θ-defensin analog RC-101 suppressed SARS-CoV-2 infection at the step of viral entry. HNP1, HD5 and RC101 also blocked infection by the Brazil P.1 and the UK B.1.1.7 variants. Linear unstructured defensins did not inhibit viral infection. Furthermore, the antiviral activity of defensins was also found for infections of epithelial cells which did not overexpress hACE2.

Our results showed that the anti-SARS-activity of defensins was specific for defensin subtypes. HBDs are generally more cationic and less hydrophobic than human α-defensins. Differential anti-SARS-CoV-2 activities among HBDs and lectin-like HNPs1–3, HD5 and RC101 suggest that hydrophobic binding properties of these defensins may play a critical role in their viral inhibition. ProHD5 exhibits reduced lectin-like properties, which is consistent with the fact that the pro-region functionally inhibits defensin activity, and presumably contributes to the loss of antiviral activity [34]. We also found differential anti-SARS-CoV-2 activities between HD5 and HD6 (Figure 1), which is not surprising as these two intestinal defensins exhibit distinct structures and properties, particularly in the case of glycan-mediated viral attachment [7,41].

Detailed mechanisms of defensin-mediated inhibition of SARS-CoV-2 fusion remain to be determined. Analyses of specific steps during viral entry revealed that HNP1 had a weak inhibitory effect on viral attachment and no effect on the binding of the spike RBD domain to hACE2, but significantly inhibited SARS-CoV-2 spike protein-mediated viral fusion. Defensins have also been shown to block viral fusion of HIV [13,42], but specific mechanisms of action were not reported. Defensins may alter protein domains of spike proteins important for viral fusion [43]. Additionally, these peptides may suppress viral entry by aggregating virions [44] or by affecting the lipid bilayer structure of the viral membrane.

Several host factors including TMPRSS2 and TMPRSS4, neutrophilin-1 and heparan sulfate interact with spike proteins and promote SARS-CoV-2 infection [45,46,47,48,49,50]. Thus, defensins may inhibit viral entry by acting on these host factors. For example, serine protease TMPRSS2 has been shown to prime SARS-CoV-2 proteins for hACE2-dependent viral entry [45]. Since RC101 is known to inhibit Japanese encephalitis virus protease [51], investigations into the effects of defensins on serine protease are warranted. Additionally, alternative receptors including CD147 and AXL for SARS-CoV-2 viral entry have been reported, particularly in cells with a low abundance of hACE2 [28,52,53]. We have shown that pseudotyped viruses expressing SARS-CoV-2 spike proteins enter A549 cells through CD147 in a spike RBD-independent manner [28]. Similarly, AXL mediates SARS-CoV-2 infection of lung epithelial cells through interactions with the N-terminal domain of SARS-CoV-2 spike proteins [53]. We found that HNP1 did not impact the binding of RBD to hACE2 but suppressed pseudotyped SARS-CoV-2 infection of A549 cells, suggesting that defensins may interfere with the binding of spike proteins to alternative receptors such as CD147.

Defensins are known for exhibiting both pro-inflammatory and anti-inflammatory activities, and they can suppress viral infection by modulating immune cell activities [14,19]. Therefore, although HBDs did not block viral entry, these peptides may inhibit viruses by promoting host restriction factors in specific cell types. Studies on the role of defensins in the regulation of viral infection through immune modulation using innate immune cells will offer insights into immune-modulatory functions of defensins relevant to SARS-CoV-2 infection and disease outcomes.

Defensins represent one category of antimicrobial peptides [54]. Antimicrobial peptides are evolutionarily conserved molecules for defense, which are produced by bacteria, insects, plants and vertebrates [54,55]. More than 3000 antimicrobial peptides have been reported [56]. Considering their versatile properties including anti-microbial activities and immune functions, the development of natural or synthetic antimicrobial peptides against SARS-CoV-2 and other viruses may help combat emerging pathogens in the future.

Our findings have clinical relevance. Individuals differ in their defensin repertoire, which may influence disease outcomes in response to SARS-CoV-2 infection [57,58,59,60]. Older adults and people with hypertension have dysfunctional neutrophils [61,62,63,64], and they have higher mortality in response to SARS-CoV-2 infection [65,66,67,68]. It is possible that in these high-risk groups, defensin levels are insufficient to block virus replication during acute infection and are insufficient to control inflammation mediated by phagocytes in the aftermath of infection, which may provide a partial explanation for severe disease outcomes in these groups. Furthermore, HNPs form stable complexes with lipoprotein (a) and low-density lipoproteins, which are elevated in people with hypertension [69]. Thus, studies on the effects of lipoproteins on HNP-mediated SARS-CoV-2 inhibition will likely offer insights into COVID-19 disease pathogenesis.

In summary, our findings provide insights into the function of human α-defensins in innate immunity against SARS-CoV-2 infection. Our findings show that α-defensins and their analog RC101 are inhibitors of SARS-CoV-2 infection. Understanding the mechanisms by which defensins and their analogs block SARS-CoV-2 infection offers new therapeutic avenues.

## Data Availability

Not applicable.

## Supplementary Materials

The following are available online at https://www.mdpi.com/article/10.3390/v13071246/s1, Figure S1: Defensins with anti-SARS-CoV2 activity are not cytotoxic.
