# Supplementary material for: Human Defensins Inhibit SARS-CoV-2 Infection by Blocking Viral Entry
Source: Viruses. 2021 Jun 26;13(7):1246. doi: 10.3390/v13071246 (PMC8310277; doi:10.3390/v13071246)
Supplement: Supplementary file 1 [file viruses-13-01246-s001.zip › viruses-1251928-supplementary.pdf]

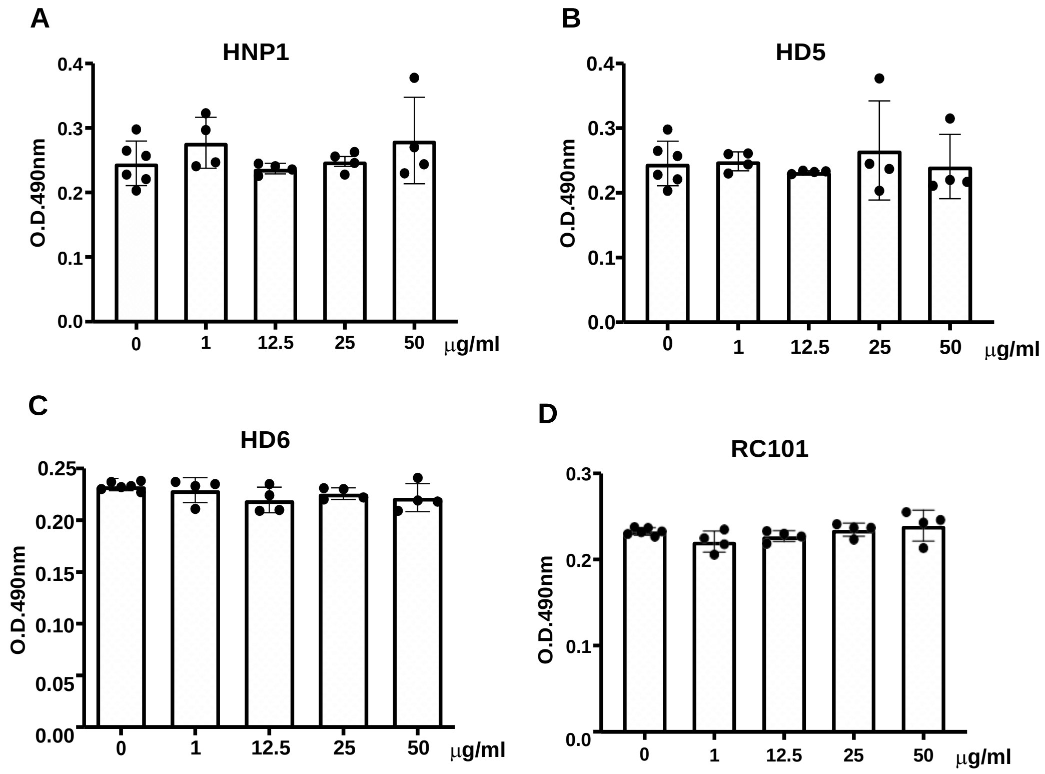

Fig S1. Defensins with anti-SARS-CoV2 activity are not cytotoxic. hACE2-expressing HEK293T cells were treated for 24 hours with the indicated concentration of HNP1 (A), HD5 (B), HD6 (C), and RC101 (D). Cell viability was determined by MTS-based CellTiter 96® AQueous One Solution Cell Proliferation Assay. Data are mean ± SD of 4-6 samples, and are representative of two independent experiments.
